# Histomorphometric and Micro-CT Evaluation of Cerabone and Bio-Oss in Maxillary Sinus Lifting: A Randomized Clinical Trial

**DOI:** 10.3390/medicina60111834

**Published:** 2024-11-08

**Authors:** Rodrigo dos Santos Pereira, Marcus Vinicius Neumann Brandão de Carvalho, Eduardo Hochuli-Vieira, Cristian Statkievicz, Déborah Laurindo Pereira Santos, Renato Torres Augusto Neto, Carolina de Fátima Soares Pinto, Francesco Bennardo, Carlos Fernando Mourão

**Affiliations:** 1 Department of Oral & Maxillofacial Surgery, University of Grande Rio—UNIGRANRIO, Rio de Janeiro 25071-202, Brazil; dr.pereira@live.com (R.d.S.P.); neumann7br@gmail.com (M.V.N.B.d.C.); carolinapintodds@gmail.com (C.d.F.S.P.); 2 Department of Diagnostic and Surgery, Araraquara School of Dentistry, Sao Paulo State University, Sao Paulo 14801-385, Brazil; eduardo.hochuli@unesp.br; 3 Department of Diagnostic and Surgery, Araçatuba School of Dentistry, Sao Paulo State University, Sao Paulo 16066-840, Brazil; c.statkievicz@gmail.com; 4 Department of Oral Surgery, Maurício de Nassau Dental School–Uninassau, Maceió 57051-565, Brazil; deborahlpsantos@gmail.com; 5 School of Dentistry, Federal University of Alagoas—UFAL, Maceió 57072-970, Brazil; rtorresneto@gmail.com; 6 School of Dentistry, Magna Graecia University of Catanzaro, Catanzaro 88100, Italy; 7 Department of Basics and Clinical Translational Sciences, Tufts University School of Dental Medicine, Boston, MA 02111, USA; carlos.mourao@tufts.edu

**Keywords:** xenograft, maxillary sinus, Bio-Oss, Cerabone

## Abstract

*Background and Objectives*: The loss of teeth in the posterior maxillary region often leads to significant alveolar bone resorption and maxillary sinus pneumatization, complicating dental implant placement. Maxillary sinus grafting, typically using autogenous bone, is a common solution. However, autogenous bone grafts require additional surgical procedures, leading to increased morbidity. This study aims to compare the efficacy of two xenografts, Bio-Oss and Cerabone, in promoting new bone formation in maxillary sinus grafting through histomorphometric analysis and micro-computed tomography (micro-CT). *Materials and Methods*: A total of 22 maxillary sinuses (12 right and 10 left) were grafted, with 12 using Cerabone and 10 using Bio-Oss. Six months post-grafting, biopsies were collected for histomorphometric analysis to measure new bone formation, connective tissue, and residual biomaterial. Additionally, micro-CT analysis was performed to assess bone volume fraction, trabecular thickness, number, and separation. *Results*: Histomorphometric analysis showed that the Cerabone group had a higher average new bone formation (25.94% ± 10.55) compared to the Bio-Oss group (17.29% ± 4.61), with a statistically significant difference (*p* = 0.02). Micro-CT analysis revealed that the bone volume fraction in the Cerabone group was significantly higher compared to the Bio-Oss group, with significant differences in trabecular thickness (*p* = 0.02) but not in trabecular number or separation. *Conclusions*: The study demonstrates that both xenografts are effective in promoting new bone formation in maxillary sinus grafting. However, Cerabone showed superior performance in terms of new bone formation and bone volume fraction, suggesting it may be a more effective option for maxillary sinus augmentation procedures.

## 1. Introduction

The utilization of dental implants has become an increasingly common resource in the process of oral rehabilitation [1]. The loss of teeth leads to significant resorption of the alveolar bone due to the absence of biomechanical stimulation. This condition is further exacerbated in the posterior maxillary region, where the maxillary sinus undergoes a physiological process of pneumatization, which can prevent dental implant placement or displace the implant into the cavity due to the lack of ideal bone height [2,3]. In such cases, one of the most commonly used methods for re-establishing bone height is maxillary sinus grafting [4,5].

Autogenous bone, regarded as the gold standard for sinus grafting, is still considered the most satisfactory bone substitute due to its osteogenic, osteoconductive, and osteoinductive properties [6,7,8]. However, its limitations include the necessity for an additional surgical procedure for harvesting, which patients often report as a source of morbidity [9]. This additional surgical step can lead to increased patient discomfort and a higher risk of complications, thus highlighting the need for alternative grafting materials.

The use of biomaterials, both synthetic and natural, has been explored as alternatives to autogenous bone. Among these, bioactive glasses, xenografts, allografts, and alloplastic grafts have shown promise [10,11,12,13,14,15]. Their clinical responses have been satisfactory, particularly due to their osteoconductive properties that facilitate the formation of scaffolds, allowing the infiltration of bone precursor cells [11,16]. These properties make them viable options for bone regeneration procedures, offering a balance between efficacy and patient comfort.

Among the most commonly used biomaterials in alveolar ridge reconstruction surgery, hydroxyapatite xenografts derived from bovine sources stand out due to their lower volumetric loss, ability to form new bone, particle interconnectivity, and load-sharing capacity with the host [17,18,19,20,21,22]. Commercially, Bio-Oss^®^ and Cerabone^®^ are prominent. They differ in their production processes: Bio-Oss is heated to 300 °C followed by a NaOH bath, while Cerabone is sintered at temperatures exceeding 1200 °C. As a result, Cerabone exhibits crystalline hydroxyapatite characteristics with porosity similar to human bone [16,23,24]. These differences in production processes can influence the material properties and, consequently, the clinical outcomes.

Xenografts have emerged as a prominent alternative to autogenous bone in maxillary sinus floor elevation surgery, offering comparable outcomes regarding new bone formation and implant survival rates [18]. The performance of xenografts can vary significantly based on their origin, processing methods, and physicochemical properties, influencing their osteoconductive potential, resorption rates, and ability to integrate with host bone [21]. While some xenografts promote new bone formation, others are superior in maintaining graft volume over time [18,21]. The addition of biological factors to xenografts has shown mixed results in enhancing their regenerative potential. This variability in outcomes underscores the importance of understanding the specific characteristics of each xenograft material and how they influence the healing process in maxillary sinus augmentation procedures, enabling clinicians to optimize xenograft selection for successful outcomes in maxillary reconstruction [16,17,18,19,20,21,22,23,24].

The objective of the present study is to evaluate, through histomorphometric analyses and micro-computed tomography (micro-CT), the new bone formation of Bio-Oss and Cerabone in human maxillary sinuses. The clinical significance of this study is in its potential to improve the outcomes of maxillary sinus grafting procedures, which are a common and challenging aspect of oral rehabilitation. This research rigorously compares the effectiveness of two widely used xenografts, Bio-Oss and Cerabone, in promoting new bone formation.

The null hypothesis (H_0_) is that there will be no significant difference in the amount of new bone formed in maxillary sinuses grafted with Cerabone after six months of bone repair. This hypothesis sets the foundation for a rigorous evaluation of the comparative efficacy of these two biomaterials in clinical practice.

## 2. Materials and Methods

This study was registered and approved on 5 July 2023, through Plataforma Brasil, which is the official clinical trial registration system of the Brazilian Ministry of Health. The protocol number for this study is 67935823.7.0000.5283. Additionally, the clinical trial has been registered with the Brazilian Register for Clinical Trials (ReBEC) under the number RBR-487ggmz.

### 2.1. Study Design and Ethical Approval

**Figure 1:**
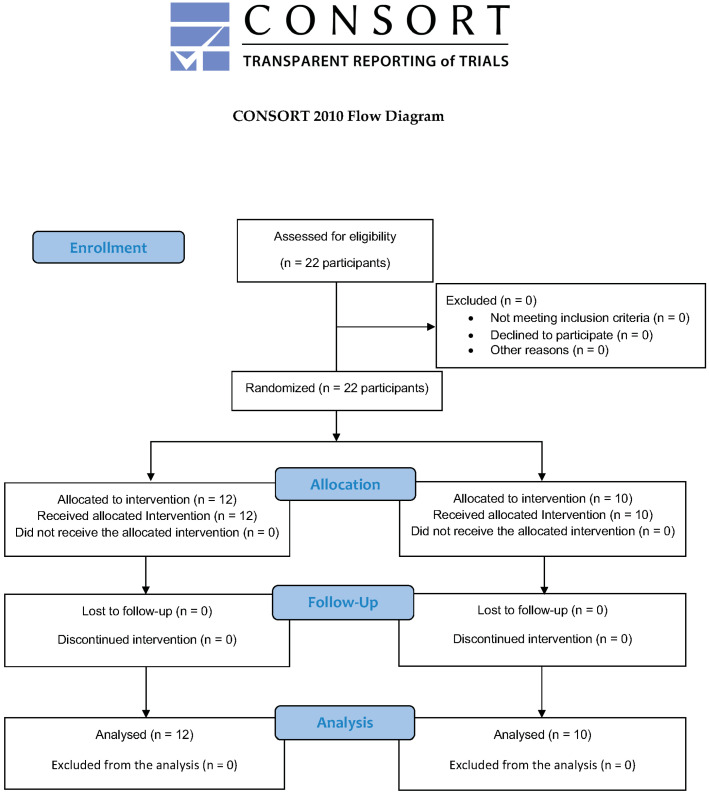
CONSORT flow diagram.

Following ethical approval, patients were invited to participate in the study at the dental clinic of the Faculty of Dentistry, UNIGRANRIO, between March 2023 and July 2024. Those who agreed to participate signed an informed consent form. The study adhered to the CONSORT guidelines to ensure the quality of the evaluation [25] (Figure 1).

### 2.2. Sample Size Determination

The minimum number of maxillary sinuses required for the study was determined using a power test available at http://calculoamostral.bauru.usp.br/ (accessed on 20 February 2023). A beta error of 20% and an alpha error of 5% were applied. A standard deviation of 9.9 and a mean difference of 14.8 were used, based on a previous study [26]. The study followed a one-tailed test, resulting in a minimum of 9 maxillary sinuses per group.

### 2.3. Inclusion and Exclusion Criteria

Inclusion criteria:

- Participants aged 18–75 years requiring reconstruction of the posterior maxillary bone height for posterior dental implant placement.
- Residual bone height in the maxillary sinus floor of less than 5 mm, as determined by prior cone-beam computed tomography (CBCT) and measured using OsiriX software version 4.1.2 (OsiriX Foundation, Geneva, Switzerland).
- Male and female participants in good general health.
- Participants with no contraindications for minor oral surgical procedures and who are committed to maintaining good oral hygiene were eligible.

Candidates needed to provide informed consent, comply with study protocols, and have no previous bone grafts in the intended augmentation area. Psychological readiness for the procedure and follow-up was also required.

Exclusion criteria:

- Participants with uncontrolled periodontal disease.
- Presence of uncontrolled systemic diseases.
- Presence of sinus pathologies.
- Smoking habits.
- Residual roots in the maxillary sinus.
- People who had undergone head and neck radiation therapy for cancer treatment.

Additionally, individuals taking medications affecting bone metabolism, pregnant or breastfeeding women, those with a history of alcohol or drug abuse, severe bruxism, osteoporosis or other metabolic bone diseases, bleeding disorders, immune deficiencies or on immunosuppressive therapy, poor oral hygiene, and psychiatric disorders impairing compliance with study protocols were excluded. Furthermore, if sinus membrane perforation and infection occurred after the surgical procedure, the patient was excluded as well.

### 2.4. Randomization

The selection of the biomaterial to be grafted in each maxillary sinus was conducted by a clinical assistant using the website “https://www.random.org” (accessed on 20 February 2023).

### 2.5. Groups Evaluated

After meeting the inclusion and exclusion criteria, the volunteers were allocated into two groups:

Group 1 (G1): 12 maxillary sinuses grafted with Cerabone^®^ (Botiss Biomaterials GmbH, Zossen, Germany).

Group 2 (G2): 10 maxillary sinuses grafted with Bio-Oss^®^ (Geistlich Pharma AG, Wolhusen, Switzerland).

### 2.6. Surgical Procedure

All procedures were performed at the dental clinic of the Faculty of Dentistry, UNIGRANRIO. The access to the maxillary sinus was performed under local anesthesia using lidocaine with adrenaline 1:100,000 (DFL—Jacarepaguá/RJ) or mepivacaine with adrenaline 1:100,000 (DFL—Jacarepaguá/RJ) for allergic patients. A crestal incision was made on the maxillary bone with a #15 blade (Solidor^®^—Barueri, SP, Brazil) to expose the lateral bony wall. A spherical diamond bur #8 (KG Sorensen—Cotia, SP, Brazil) was used under copious irrigation with 0.9% saline solution (ADV—Nova Odessa, SP, Brazil) to create a fenestration for access to the sinus membrane. Sinus membrane elevators (Neodent^®^—Curitiba, Brazil) were used to carefully elevate the membrane, and the proposed bone substitute was grafted. Wound closure was performed with 4-0 polyglactin absorbable sutures (Ethicon^®^—São Paulo, Brazil) (27).

### 2.7. Postoperative Care

Postoperatively, patients were prescribed 500 mg of amoxicillin (EMS, São Paulo, SP, Brazil) three times a day and a rinse of chlorhexidine digluconate 0.12% twice per day for seven days. Additionally, 750 mg of paracetamol (São Paulo, SP, Brazil) was prescribed every six hours for the first 48 h and continued as needed for pain management.

### 2.8. Biopsy Collection and Laboratory Procedures

Biopsies were collected after six months of bone healing during the dental implant placement. Samples were harvested using a surgical guide and a 3.0 × 15 mm trephine bur (MK Life, Porto Alegre, RS, Brazil) and stored in 10% formalin solution (pH 7) for 24 h, maintaining apical orientation. After 48 h in formaldehyde, biopsies were washed in running water for 24 h and embedded in paraffin. Paraffin blocks were sectioned at 5 μm thickness and stained with hematoxylin and eosin (H&E).

### 2.9. Histomorphometric Analysis

Four sections from each biopsy were stained with hematoxylin and eosin (H&E) for the morphometric analysis of new bone tissue, connective tissue, and residual biomaterial in the maxillary sinuses. Sections were coded into three areas: bed, intermediate, and apical, according to Pereira et al. [27], and examined under a light microscope at 10× magnification by a single evaluator, excluding the recipient bed from analysis. Images were captured with a microscope equipped with a camera (Leica DM 500) and analyzed using Image J 153t software (National Institutes of Health, Bethesda, MD, USA). Areas (μm^2^) of remaining particles, connective tissue, and new bone formation were measured, and results were converted to percentages for each evaluated structure.

### 2.10. Micro-Computed Tomography Analysis (Micro-CT)

Samples were washed in running water for 24 h and stored in 70% alcohol before micro-CT analysis (SkyScan 1174; Bruker microCT, Kontich, Belgium) in horizontal orientation, maintaining apical–coronal orientation. Samples were fixed in the device tubes and sectioned at 8.74 μm thickness with 50 kV X-ray energy and a 500 mA current. Images were captured with a pixel size of 7.67 μm, with 1024 line counts and 1277 column counts. The rotation step was 0.3° over 360° with an average frame count of 3. An aluminum filter (0.25 mm) was used, and the scan duration averaged 1 h and 13 min.

Images were reconstructed using NRecon v1.6.9.8 software (Bruker microCT, Kontich, Belgium) with a smoothing factor of 5, a ring artifact correction of 5, a beam hardening correction of 20%, and an image correction range of 0.0 to 0.11. Reconstructed 3D images were analyzed using CT-analyzer (CTAn) v1.12.4.0 software (Bruker microCT, Kontich, Belgium). Images were aligned axially, and 100 slices from the apical region were selected using the region of interest (ROI) tool in a circular format (1.5 × 1.5 mm). Intact bone was considered the most cortical bone based on CBCT-determined bone height. The selected area included only the bone graft throughout the circumference, excluding the intact bone region. Broken samples were excluded from analysis. Using the binary selection visualization tool, the histogram was standardized at 255% with a 70 index. Morphometric analysis was performed to assess 3D trabecular bone data, including bone volume fraction (BV/TV), trabecular thickness (Tb.Th), trabecular number (Tb.N), and trabecular separation (Tb.Sp), as reported by Bouxsein et al. [28]. All analyses and data collection were conducted by a single trained researcher.

### 2.11. Statistical Analysis

Statistical tests were performed using GraphPad Prism 8 (San Diego, CA, USA) software. The Shapiro–Wilk test was used to determine the homoscedasticity of the samples. Parametric data were evaluated using Student’s *t*-test, and non-parametric data were analyzed using the Mann–Whitney test. A *p*-value of ≤0.05 was considered significant.

## 3. Results

A total of twenty-two patients (9 men and 13 women), aged between 41 and 75 years (mean age 56.6 years, median age 54 years, and standard deviation 8.7 years), underwent unilateral maxillary sinus augmentation using the two types of bone grafts proposed in this study.

The present study included patients with the following self-declared ethnicities: mixed race or “Brazilian pardo” (G1: 8; G2: 3), White (G1: 2; G2: 5), Black (G1:1; G2: 2), and Asian (G1: 1, G2: 0).

Regarding relevant medical history, 27% (*n* = 6) of patients had controlled hypertension, 18% (*n* = 4) had well-managed type 2 diabetes, and 9% (*n* = 2) had osteopenia. None of the patients had a history of osteoporosis or other bone metabolism disorders.

The primary reasons for seeking sinus lift procedures were single tooth replacement (45%, *n* = 10), multiple tooth replacement (41%, *n* = 9), and full arch rehabilitation (14%, *n* = 3). The average duration of tooth loss prior to the procedure was 3.7 years (range 1–10 years). Five patients (23%) had previous experience with dental implants in other areas of their mouths.

All patients underwent a comprehensive oral examination and a cone-beam computed tomography (CBCT) scan prior to the procedure. The average residual bone height in the maxillary sinus floor was 3.2 mm (range 1.5–4.8 mm).

### 3.1. Group 1: Cerabone^®^ Graft

**Figure 2:**
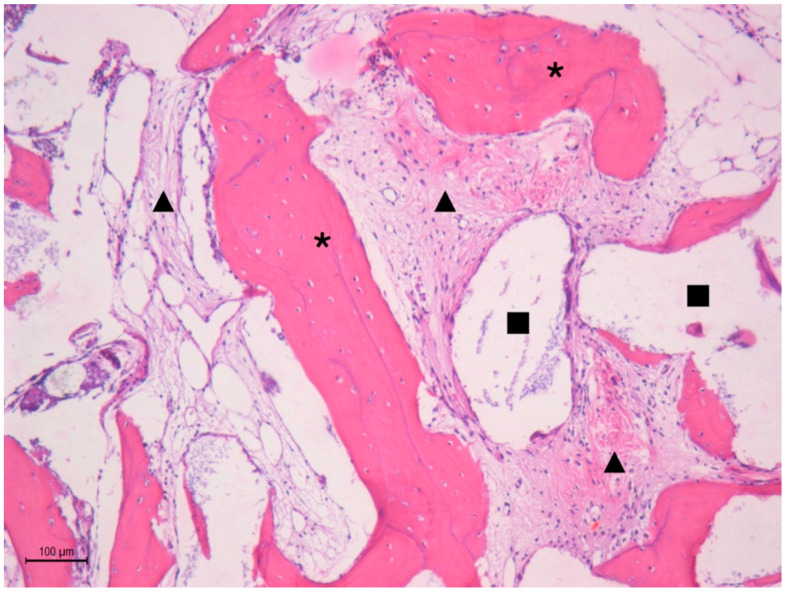
Histological image of Group 1 showing the new bone formed (*), connective tissue (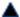), and Cerabone particle (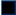).

In Group 1, new bone formation exhibited a lamellar structure, with osteocytes embedded in the bone matrix and osteoblasts present at the periphery. Additionally, a well-cellularized connective tissue was formed, along with significant residual biomaterial particles showing adjacent bone formation. The average percentage of new bone formation was 25.94% ± 10.55, that of connective tissue was 51.00% ± 10.31, and the median for remaining biomaterial was 20.34%. These results are summarized in Figure 2 and Table 1.

### 3.2. Group 2: Bio-Oss^®^ Graft

**Figure 3:**
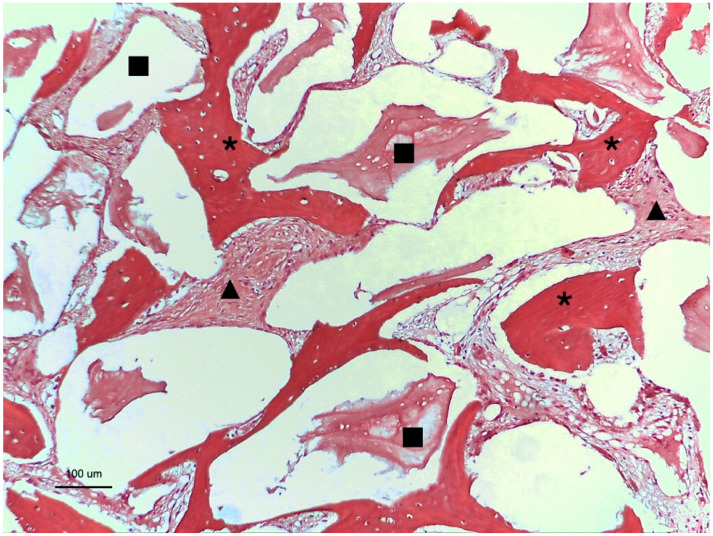
Histological image of Group 2 showing the new bone formed (*), connective tissue (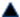), and Bio-Oss particles (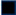).

**Figure 4:**
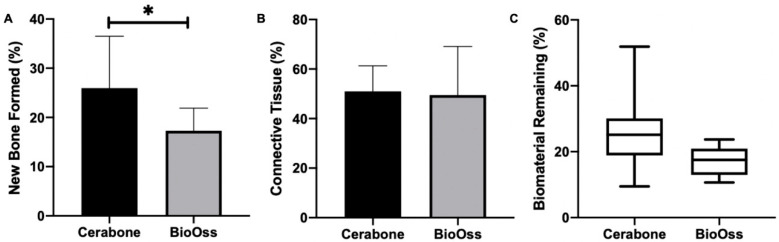
Graphics demonstrating the results for new bone formation (**A**), connective tissue (**B**), and biomaterial remining (**C**). * indicates statistical difference (*p* > 0.05).

In Group 2, the morphological characteristics were similar to those observed in Group 1, with new bone formation being well cellularized, as well as the connective tissue and residual biomaterials showing adjacent bone formation. The percentage of new bone formation was 17.29% ± 4.61, that of connective tissue was 49.46% ± 19.69, and the median for remaining biomaterial was 38.10%. These results are detailed in Figure 3 and Figure 4A–C and Table 2.

There was a statistically significant difference between the groups for new bone formation (*p* = 0.02), but not for connective tissue (*p* = 0.81) or residual biomaterial (*p* = 0.31).

### 3.3. Micro-CT Analysis

**Figure 5:**
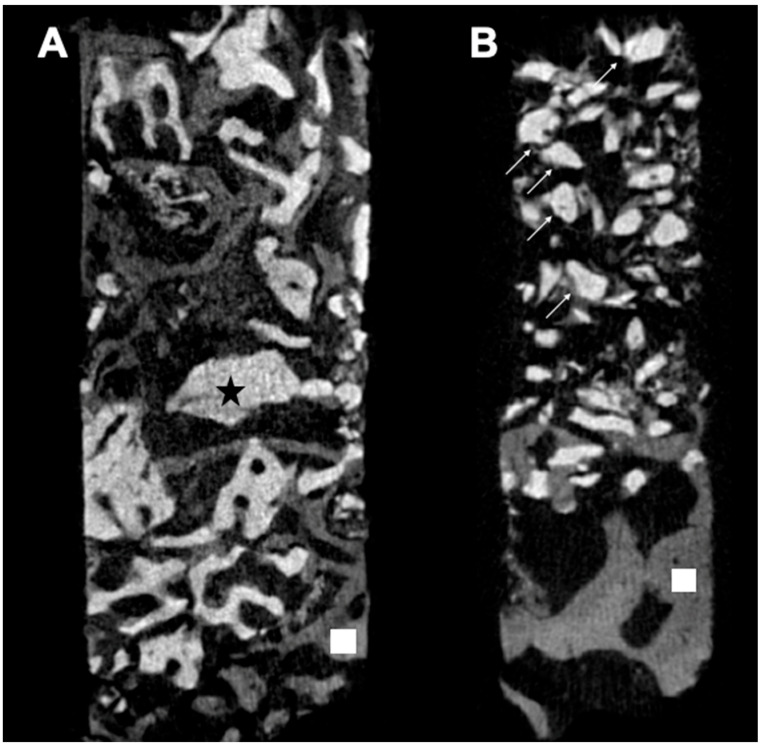
Micro-CT reconstruction image of a coronal section for Groups 1 (**A**) and 2 (**B**) showing the pristine bone (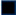), Cerabone particle (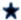), and Bio-Oss particle (→).

**Figure 6:**
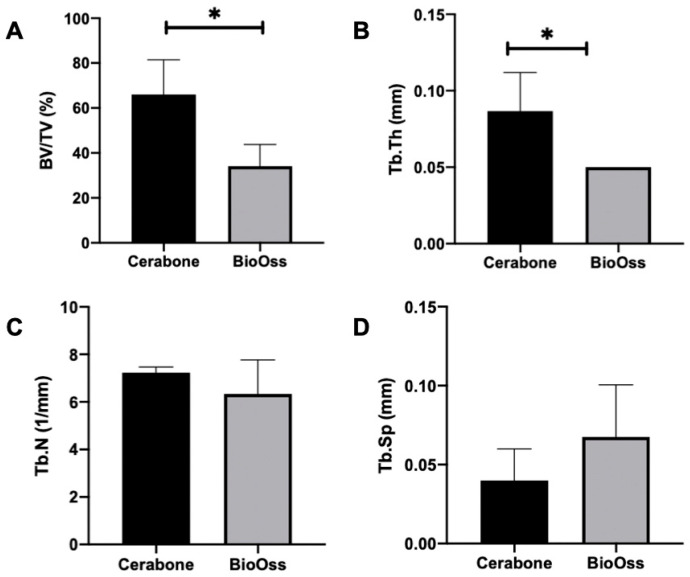
Graphics demonstrating the results for BV/TV (**A**), Tb.Th (**B**), Tb.N (**C**), and Tb.SP (**D**). * indicates statistical difference (*p* > 0.05).

Micro-CT analysis showed that the mean bone volume fraction (BV/TV) in Group 1 was 66.06%, while in Group 2, it was 34.11%, with a statistically significant difference between the groups (*p* = 0.01). The trabecular thickness (Tb.Th) in Group 1 averaged 0.08 mm, compared to 0.05 mm in Group 2, also showing a statistically significant difference (*p* = 0.02). The trabecular number (Tb.N) averaged 7.22 1/mm in Group 1 and 6.34 1/mm in Group 2 (*p* = 0.34). The mean trabecular separation (Tb.Sp) was 0.04 mm in Group 1 and 0.06 mm in Group 2 (*p* = 0.26). These results are illustrated in Figure 5A,B and Figure 6A–D.

## 4. Discussion

This study’s findings show that mature bone capable of supporting dental implants for oral rehabilitation was formed. Table 1 and Table 2 contain detailed results about new bone formation, connective tissue, and remaining biomaterial for both groups. A thorough statistical analysis confirmed the significance of the observed differences, indicating the effectiveness of Cerabone^®^ in promoting new bone formation compared to Bio-Oss^®^. Thus, the null hypothesis (H_0_) was disproven.

Kacarevic et al. [23] reported that the processing of both bone substitutes evaluated in the present study results in an increase in hydroxyapatite crystal sizes, which can vary up to 300% for Bio-Oss and up to 1000% for Cerabone. Furthermore, another study highlighted that Bio-Oss demonstrated more regular particle sizes compared to other biomaterials [29].

The use of bovine hydroxyapatite has been extensively employed in the reconstruction of various alveolar ridge defects [30,31]. In procedures for maintaining alveolar ridge dimensions, Ramaglia et al. [31] reported an average bone formation of 47.76% using Bio-Oss as the bone substitute, with approximately 25.43% of the biomaterial remaining. Similar values have been found using Cerabone, with around 30.47% new bone formation [32]. Thus, both biomaterials have demonstrated satisfactory osteoconductivity and favorable biocompatibility.

When evaluating sinus augmentation, various biomaterials, such as blood clots, autogenous bone, beta-tricalcium phosphate, and bioactive glass, have shown satisfactory results in reconstruction [30,33,34,35]. The average new bone formation ranges from 27.7% to 45.6%, with histological characteristics similar to those found in the present study. However, the percentage of remaining biomaterial, particularly those with slow degradation rates, directly influences the amount of new bone formed.

Bovine-derived hydroxyapatite has been widely used for sinus bone height augmentation. Piatelli et al. [36] studied Bio-Oss and reported an average bone formation of 30%, which was also observed by Pereira et al. [7] with approximately 34.3%. Using Cerabone, Mahesh et al. [37] found an average of 39.23% new bone formation after maxillary sinus repair. The different values compared to the present study can be attributed to the methodology applied, where the area was measured in μm^2^ and then converted to a percentage. However, the histological findings are consistent with those described in the literature, such as the absence of inflammatory cells, well-vascularized and cellularized connective tissue, and lamellar bone, indicating maturity suitable for receiving dental implants [23,24,36].

Bovine hydroxyapatite is known for its extremely slow degradation rate [38]. Fleming et al. [39] compared the resorption rates of bovine hydroxyapatite and synthetic materials, showing a 5% to 15% annual decrease due to macrophage activity, corroborating Goetz et al.’s findings [12]. Bonardi et al. [30] compared beta-tricalcium phosphate (B-TCP) with Bio-Oss in sinus grafts, noting different resorption times: B-TCP is completely degraded within 18 months, while Bio-Oss maintains volume [34,38]. The methodology in their study measured 5291 μm^2^ for B-TCP and 56,258 μm^2^ for Bio-Oss, aligning with the results of the present study and explaining the lower percentage of new bone formation.

Micro-CT offers advantages such as preserving the sample’s microarchitecture and being faster, although it cannot evaluate cellular composition [40]. Autogenous bone showed BV/TV values of 57.19%, Tb.Th of 0.38 mm, Tb.N of 2.04 1/mm, and Tb.Sp of 0.38 mm [41]. The literature indicates BV/TV, Tb.Th, Tb.N, and Tb.Sp values around 37%, 0.166 mm, 2.45 1/mm, and 0.156, respectively, using Bio-Oss in maxillary sinuses after 5 months of bone repair [18]. Pereira et al. [41] studied bioactive glass in maxillary sinus reconstructions, reporting BV/TV of 52.06%, Tb.Th of 0.14 mm, Tb.N of 2.70 1/mm, and Tb.Sp of 0.13 mm. The BV/TV values were similar to the present study, although the other values differed, indicating an adaptive microarchitecture in response to new bone formation.

While this study provides valuable insights into the effectiveness of Cerabone^®^ and Bio-Oss^®^ in maxillary sinus augmentation, it has some limitations. One significant limitation is the lack of long-term follow-up data to assess the survival rates of dental implants placed in the grafted areas. Previous studies have demonstrated satisfactory survival rates for implants in areas grafted with Bio-Oss [42]; however, similar data for Cerabone are not yet available. This gap in the literature underscores the need for future clinical studies to evaluate the long-term performance and load-sharing capacity of Cerabone. Bio-Oss, despite forming less bone, is known to share load effectively with the host bone, which contributes to its documented success.

This study, while providing valuable insights into the comparative efficacy of Cerabone and Bio-Oss in maxillary sinus augmentation, has some limitations that should be considered. Firstly, the sample size, although statistically determined and sufficient for our primary outcomes, was relatively small. This may limit the generalizability of our findings to broader populations. Secondly, the unequal group sizes (12 in the Cerabone group versus 10 in the Bio-Oss group), while not compromising the statistical validity of our results, could potentially introduce bias in secondary analyses. Additionally, our follow-up period of six months, while adequate for assessing initial bone formation, does not provide insight into long-term outcomes or the survival rates of dental implants placed in the augmented sites. Lastly, while histomorphometric and micro-CT analyses provide valuable quantitative data, they do not fully capture the qualitative aspects of the clinical performance of the grafts under functional load.

Future studies with larger sample sizes, longer follow-up periods, and assessment of functional outcomes should be conducted to support and expand upon our findings. It is essential to include dental implants in masticatory functions to confirm the outcomes observed in this study and to gain a more comprehensive understanding of the clinical performance of these biomaterials. Additionally, these studies should focus on the comparative long-term effectiveness and integration of both biomaterials in diverse patient populations.

## 5. Conclusions

In conclusion, this study found that both Cerabone^®^ and Bio-Oss^®^ were histologically effective in promoting new bone formation after six months. Although Bio-Oss exhibited a lower rate of bone formation and higher residual biomaterial than Cerabone, it still effectively supported osseointegrated implants, indicating its capability to provide structural support over time. Cerabone demonstrated a higher rate of new bone formation, which is advantageous for rapid bone regeneration, while Bio-Oss maintained volume and load-sharing with host bone, which is beneficial for long-term structural integrity.

## Figures and Tables

**Table 1:** Outcomes of the new bone formed, connective tissue, and biomaterial remaining for Group 1 (Cerabone^®^).

Maxillary Sinus	Bone Formed (%)	Connective Tissue (%)	Biomaterial Remaining (%)
1R	9.49	38.05	52.47
2R	15.28	46.22	38.51
3L	2.2	72.39	5.61
4R	17.83	43.74	38.43
5L	28.38	54.54	17.08
6L	32.32	54.36	13.31
7R	25.06	51.33	23.61
8R	2.99	42.88	27.22
9L	51.89	39.12	8.99
10R	25.24	64.12	10.64
11L	30.17	58.11	11.72
12L	23.77	4.72	29.03
Mean	25.94	51.00	-
SD	10.55	10.31	-
Median	-	-	20.34

R = right maxillary sinus. L = left maxillary sinus.

**Table 2:** Outcomes of the new bone formed, connective tissue, and biomaterial remaining for Group 2 (Bio-Oss^®^).

Maxillary Sinus	Bone Formed (%)	Connective Tissue (%)	Biomaterial Remaining (%)
13R	23.37	76.67	0
14L	1.91	80.28	0.76
15L	10.68	22.16	42.17
16R	18.38	35.17	46.63
17E	16.45	25.79	37.48
18R	20.09	51.44	28.95
19L	23.72	64.01	12.24
20R	1.07	50.63	38.72
21R	16.64	41.31	42.07
22L	13.74	47.16	39.09
Mean	17.29	49.46	-
SD	4.61	19.69	-
Median	-	-	38.10

R = right maxillary sinus. L = left maxillary sinus.

## Data Availability

No new data were created or analyzed in this study. Data sharing is not applicable to this article.
